# Baseline characteristics predicting clinical outcomes and serious adverse events in middle-aged hypertensive women: a subanalysis of the SPRINT in women aged <65 years

**DOI:** 10.3906/sag-1907-144

**Published:** 2020-08-26

**Authors:** Volkan AYDIN, Ahmet AKICI, Sibel SAKARYA, Mehmet AKMAN, Ali Serdar FAK

**Affiliations:** 1 Hypertension and Atherosclerosis Research Center (HIPAM), Marmara University, İstanbul Turkey; 2 Department of Public Health, School of Medicine, Koç University, İstanbul Turkey

**Keywords:** Hypertension, middle-aged women, intensive pharmacotherapy, cardiovascular, events

## Abstract

**Background/aim:**

The predictability of clinical outcomes in hypertension in specific patient groups, especially underrepresented populations is the key to rational treatment. This study aimed to investigate the impact of baseline characteristics of <65-year-old hypertensive women with an increased risk of cardiovascular events, managed with standard- or intensive-approach, on their clinical outcomes and serious adverse events (SAEs).

**Materials and methods:**

Baseline characteristics of <65-year-old hypertensive women (n = 1247) in SPRINT, a multicenter randomized trial to compare standard and intensive antihypertensive treatment, were analyzed with Cox-regression method to determine potential predictors of the clinical outcomes and SAEs. The primary outcome was the composite of myocardial infarction (MI), non-MI acute coronary syndrome, stroke, heart failure, or cardiovascular death.

**Results:**

The primary outcome occurred in 3.1% and SAEs in 27.6% of the population. The treatment groups were similar in terms of the primary outcome, SAEs, or their individual components. The primary outcome occurred significantly more in current smokers vs. nonsmokers (HR: 2.85, 95% CI: 1.34–6.09). The subjects who were on aspirin in the intensive-group were significantly more likely to develop the primary outcome (HR: 3.17, 95% CI: 1.23-8.19) and MI (HR: 10.15, 95% CI: 1.19-86.88) compared with those not using aspirin. The risk of overall SAEs was significantly higher in blacks vs. nonblacks (HR: 1.27, 95% CI: 1.01-1.58), in current-smokers vs. nonsmokers (HR: 1.59, 95% CI: 1.23-2.05), and those with vs. without chronic kidney disease (CKD), (HR: 1.38, 95% CI: 1.08-1.77). The likelihood of SAEs significantly increased with age (HR: 1.04, 95% CI: 1.01-1.07).

**Conclusion:**

Smoking, aspirin, CKD, black race, and age seemed as important baseline characteristics in follow-up of <65-year-old hypertensive women, also depending on therapeutic strategy. Clinicians are expected to consider these critical parameters for effective antihypertensive management that promotes better outcomes in this middle-aged female population.

## 1. Introduction

Sex-related variations exist in cardiovascular (CV) anatomy and physiology, predisposing women and men at differential CV risks [1,2]. Being more vulnerable, hypertensive women have poorer CV outcomes than do men, especially in the presence of CV risk factors [3–5], despite the higher rates of awareness, antihypertensive prescription, controlled disease, and drug compliance among women [3,6]. Moreover, the observed sex-related differences in CV outcome was reported to be independent from the response to antihypertensive medications [7]. Aging further contributes to sex-related differences in developing CV disease and outcomes [8]. In fact, female predominance in hypertension takes place after the age of 65 years [6]. This is accompanied by less-controlled and more severe disease in elderly hypertensive women than their younger counterparts [9]. Hypertensive patients usually have a number of CV disease risk factors, either modifiable or relatively fixed. Targeting modifiable risk factors, total CV risk assessment contributes both to improved blood pressure (BP) control and reduced CV disease burden [10,11]. There is substantial evidence from clinical studies, where these risk factors are addressed by pre-determination of specific subgroups, helping physicians to achieve rational management of diseases in various patient subsets. Indeed, while it might not be possible to cover all clinical scenarios in a hypertension trial; underrepresentation of key demographic patient strata, like age or sex, is likely to have an impact on the generalization of the study results, e.g. predictability of clinical outcomes in such populations [12–14]. Representation of women in study populations was reported to vary from 35.2% to 59% in key hypertension trials among which the lowest rate belonged to the recent Systolic Blood Pressure Intervention Trial (SPRINT) [9]. In brief, SPRINT was a multicenter (n = 102) randomized clinical trial where 9,361 hypertensive patients at increased CV risk were assigned to either receive standard- (a target systolic BP [SBP] of <140 mmHg) or intensive-treatment (a target SBP of <120 mmHg) [15]. A recent review discussed low enrollment and fewer adverse event rates in SPRINT, implying gaps of evidence about optimal management of hypertension in women [5]. This might be more pronounced in younger women where the absolute CV risk could be regarded as low. Nevertheless, this population could also have relatively increased risk if markedly abnormal CV risk factors are present [11]. A better understanding of the key characteristics of such underrepresented populations could help to predict clinical outcomes in hypertension, and hence, contribute to the rational management of the disease. This study aimed to investigate the impact of baseline characteristics of <65-year-old hypertensive women, managed with standard or intensive approach, on their clinical outcomes and serious adverse events (SAEs) in SPRINT.

## 2. Materials and methods

### 2.1. Study population

The rationale and design of the study and eligibility of patients with their baseline characteristics in SPRINT was previously described [16]. Patients were regarded at increased CV risk if they had one or more of the following: history of clinical or subclinical CV disease, chronic kidney disease (CKD), 10-year Framingham risk score of ≥15%, or age ≥75 years. Subjects were excluded if they had diabetes mellitus, history of stroke, polycystic kidney disease, symptomatic heart failure or reduced ejection fraction <35%, estimated glomerular filtration rate (eGFR) <20 mL/min/1.73 m2 or end-stage renal disease, or were (or planned to be) pregnant [15]. In this subanalysis of the trial, we evaluated data of female population aged below 65 years old (range: 48–64 years). 

### 2.2. Clinical outcomes

The primary outcome of the SPRINT was the first occurrence of the composite of myocardial infarction (MI), non-MI acute coronary syndrome (ACS), stroke, heart failure, or CV death. Secondary outcomes included individual components of the primary composite outcome, any death, and the composite of primary endpoint or any death. We only included these clinical endpoints to our subanalysis, not evaluating renal outcomes in this subpopulation. 

### 2.3. Serious adverse events

An adverse event in the study was adjudicated to be serious if it (i) was fatal or life-threatening, (ii) led to significant or persistent disability or any congenital anomaly/birth defect, (iii) caused/prolonged hospitalization, or (iv) was judged by the investigator to pose a significant hazard or harm to the patient, requiring preventive medical or surgical intervention. Hypotension, bradycardia, electrolyte abnormalities, injurious falls, syncope, acute kidney injury or acute renal failure, or any unexpected event that the investigator believed it to be trial-related were categorized as SAEs if they met abovementioned decision-making criteria. Other adverse events not labelled as serious were not included to this subanalysis.

### 2.4. Study variables

The baseline characteristics that were used for predicting clinical outcomes and SAEs included ten variables: age, SBP, diastolic BP, race, smoking status, daily aspirin use, body mass index (BMI), total cholesterol (TC)/high-density lipoprotein cholesterol (HDL-C) ratio, hyperglycemia, and CKD. Among categorical variables, race was self-reported as black or nonblack. Smoking status was grouped as nonsmokers, former smokers, and current smokers. BMI groups were divided into three as under-/normoweight (<25 kg/m2), overweight (25 to <30 kg/m2), and obese (≥30 kg/m2). The cut-off point for TC/HDL-C ratio was chosen as 3.5 where the values above this were reported to be indicative of CV risk among women [17]. The patients who had a blood glucose level of ≥100 mg/dL at baseline were categorized as hyperglycemic [18]. CKD was defined as having an eGFR of 20–60 mL/min./1.73 m2.

### 2.5. Statistical analysis

Study data were analyzed using SPSS version 25.0 software package. Descriptive baseline and outcome data were expressed as the mean (± standard deviation) or percentage of the population. Independent t-test were used for comparing parametric variables, and either chi-square or Fisher’s exact test, where appropriate, for comparing categorical variables between standard- and intensive-treatment groups. The abovementioned baseline characteristics were entered into the Cox proportional hazards regression with a backward selection to determine independent predictors of defined outcomes. The Cox regression was performed for the total population and for each of the standard and intensive-treatment groups separately. Only those characteristics in the last steps that were regarded as statistically significant predictor of the given outcome in the standard- or intensive-treatment groups, or in overall were mentioned in the tables for concise demonstrative purposes. Hazard ratio (HR) was calculated with a 95% confidence interval (CI). An overall 5% type-I error level was used to infer statistical significance.

## 3. Results

We identified 1247 women who were below 65 years old representing 13.3% of overall SPRINT cohort, where the median duration of follow-up was 3.25 years. The baseline characteristics of this subgroup randomized to either standard-treatment (n = 595) or intensive-treatment (n = 652) were similar except slightly higher percentage of women with increased TC/HDL-C ratio in the latter (Table 1). The primary outcome event occurred in 21 (3.5%) and 18 patients (2.8%) in standard-treatment and intensive-treatment groups, respectively. On the other hand, a total of 341 SAEs were detected; 27.6% in the standard and 27.1% in the intensive strategy. The groups did not differ in terms of clinical or safety outcomes or their individual components. 

**Table 1:** Baseline characteristics of the study groups.

	Standard-treatment n = 595	Intensive-treatment n = 652
Age, y (mean ± SD)	58.4 ± 4.0	58.6 ± 3.9
Black origin, %	60.5	58.0
Body mass index category		
Under-/normoweight, %	12.7	14.2
Overweight, %	29.5	25.6
Obese, %	57.8	60.2
Systolic blood pressure, mmHg (mean ± SD)	140.1 ± 17.4	140.4 ± 17.3
Diastolic blood pressure, mmHg (mean ± SD)	83.8 ± 11.9	83.9 ± 11.4
Smoking		
Never, %	49.3	49.1
Former, %	23.4	22.8
Current, %	27.3	28.1
Hyperglycemia (≥100 mg/dL), %	39.5	37.4
TC/HDL-C ratio ≥3.5, %*	59.0	65.3
CKD (eGFR < 60 mL/min/1.73 m2), %	20.5	21.0
Aspirin use, %	35.8	33.5

*P = 0.02; CKD: Chronic kidney disease; eGFR: Estimated glomerular filtration rate; TC/HDL-C: Total cholesterol/high-density lipoprotein cholesterol.

### 3.1. Clinical outcomes

The primary outcome event occurred significantly more in current smokers vs. nonsmokers (HR: 2.85, 95% CI: 1.34–6.09). While no such effect was found in the standard-treatment group, this effect was also significantly shown in the intensive-treatment group consistent with those of the overall women population. The subjects who were on aspirin at randomization in the intensive-treatment group were significantly more likely to develop the primary outcome (HR: 3.17, 95% CI: 1.23–8.19) and MI (HR: 10.15, 95% CI: 1.19–86.88) compared with those who were not using aspirin. This association was not detected in the standard-treatment group or in overall population (Table 2).

**Table 2:** Predictors of clinical outcomes by treatment groups.

	Total		Standard-treatment		Intensive-treatment	
Clinical outcomes*	HR (95% CI)	P-value	HR (95% CI)	P-value	HR (95% CI)	P-value
SPRINT primary outcome (n = 39)						
Current vs. no smoking	2.85 (1.34–6.09)	0.007	2.54 (0.93–6.92)	0.068	3.86 (1.16–12.84)	0.028
Aspirin vs. no aspirin use	1.61 (0.86–3.03)	0.137	0.91 (0.35–2.34)	0.852	3.17 (1.23–8.19)	0.017
Myocardial infarction (n = 14)						
Aspirin vs. no aspirin use	1.91 (0.67–5.44)	0.227	0.59 (0.12–2.91)	0.514	10.15 (1.19–86.88)	0.034
Nonmyocardial infarction ACS (n = 6)						
Hyperglycemia vs. normoglycemia	9.59 (1.05–87.59)	0.045	n/a	0.932	n/a	0.770
Heart failure (n = 11)						
Age at randomization	0.92 (0.79–1.06)	0.246	1.02 (0.83–1.24)	0.875	0.69 (0.49–0.96)	0.026
CKD vs. no CKD (eGFR ≥ 60 mL/min/1.73 m^2^)	6.48 (1.89–22.19)	0.003	5.31 (1.19–23.75)	0.029	21.31 (0.54–841.16)	0.103
Death from any cause (n = 17)						
Current smoking vs. no smoking	6.46 (1.75–23.81)	0.005	15.26 (1.79–130.15)	0.013	3.26 (0.53–19.88)	0.201
CKD vs. no CKD (eGFR ≥ 60 mL/min/1.73 m^2^)	3.50 (1.31–9.37)	0.012	9.74 (2.66–35.59)	0.001	0.58 (0.07–4.91)	0.613
SPRINT primary outcome or any death (n = 54)						
Former smoking vs. no smoking	2.27 (1.07–4.84)	0.033	1.96 (0.69–5.58)	0.209	2.73 (0.92–8.12)	0.072
Current smoking vs. no smoking	4.15 (2.13–8.08)	0.000	5.00 (2.04–12.25)	0.000	3.50 (1.29–9.49)	0.014
Aspirin vs. no aspirin use	1.49 (0.87–2.54)	0.148	1.11 (0.51–2.42)	0.793	2.38 (1.06–5.31)	0.035
CKD vs. no CKD (eGFR ≥ 60 mL/min/1.73 m^2^)	1.96 (1.10–3.52)	0.023	3.65 (1.75–7.61)	0.001	0.68 (0.54–2.05)	0.497

* Stroke (n = 9) and cardiovascular death (n = 2) were not included to the table as Cox regression yielded no significant predictor in any of the treatment arms or in overall. ACS: Acute coronary syndrome; CKD: Chronic kidney disease; eGFR: Estimated glomerular filtration rate.

Among other individual components of the primary outcome, non-MI ACS was found significantly more in women with hyperglycemia than those with normoglycemia (HR: 9.59, 95% CI: 1.05–87.59). Heart failure occurred significantly more in the presence of CKD in overall (HR: 6.48, 95% CI: 1.89–22.19) and in the standard-treatment group (HR: 5.31, 95% CI: 1.19–23.75), whereas this effect disappeared in the intensive-treatment group. On the other hand, those patients who were older at randomization developed less heart failure in the intensive-treatment group (HR: 0.69, 95% CI: 0.49–0.96), an effect which was not shown in the standard-arm or overall. No predictors of risk were detected in terms of stroke or CV death (Table 2).

Death from any cause was found to occur significantly more in current smokers vs. nonsmokers (HR: 6.46, 95% CI: 1.75–23.81) and in women with vs. without CKD (HR: 3.50, 95% CI: 1.31–9.37). While similar effects were also consistently observed in the standard-treatment group, no such significant associations were detected in the intensive-arm (Table 2).

Primary outcome event or death occurred significantly more in former (HR: 2.27, 95% CI: 1.07–4.84) or current (HR: 4.15, 95% CI: 2.13–8.08) smokers vs. nonsmokers. The risk was also significantly higher in patients with vs. without CKD (HR: 1.96, 95% CI: 1.10–3.52). The subjects who were under aspirin in the intensive-treatment group were more likely to develop the primary outcome or death compared with patients who were not using aspirin at baseline (HR: 2.38, 95% CI: 1.06–5.31), (Table 2). 

### 3.2. Serious adverse events

The risk of overall SAEs was significantly higher in those with black vs. nonblack origin (HR: 1.27, 95% CI: 1.01–1.58), in current smokers vs. nonsmokers (HR: 1.59, 95% CI: 1.23–2.05), and those with vs. without CKD (HR: 1.38, 95% CI: 1.08–1.77). While these risks were also observed in the standard-treatment group, no such association was found in the intensive-treatment group. In addition, the likelihood of SAE significantly increased with the age (HR: 1.04, 95% CI: 1.01–1.07), and the effect was also observed to disappear in the intensive-treatment group (Table 3).

**Table 3:** Predictors of serious adverse events by treatment groups.

	Total		Standard-treatment		Intensive-treatment	
Serious adverse events	HR (95% CI)	P-value	HR (95% CI)	P-value	HR (95% CI)	P-value
Any (n = 341)						
Age at randomization	1.04 (1.01–1.07)	0.012	1.06 (1.01–1.10)	0.005	1.02 (0.97–1.06)	0.475
Black vs. nonblack ethnicity	1.27 (1.01–1.58)	0.037	1.63 (1.16–2.28)	0.005	1.05 (0.77–1.43)	0.740
Current smoking vs. no smoking	1.59 (1.23–2.05)	0.000	1.81 (1.25–2.61)	0.002	1.33 (0.94–1.88)	0.105
CKD vs. no CKD (eGFR ≥ 60 mL/min/1.73 m^2^)	1.38 (1.08–1.77)	0.011	1.83 (1.30–2.58)	0.001	1.06 (0.74–1.52)	0.741
Related (n = 22)						
Black vs. nonblack ethnicity	5.44 (1.59–18.60)	0.007	n/a	0.958	2.83 (0.78–10.20)	0.111
Current smoking vs. no smoking	3.55 (1.22–10.30)	0.020	5.18 (0.82–32.88)	0.081	2.54 (0.64–10.14)	0.186
CKD vs. no CKD (eGFR ≥ 60 ml/min/1.73 m^2^)	3.76 (1.58–8.97)	0.003	6.27 (1.30–30.30)	0.022	3.47 (1.11–10.85)	0.033
Hypotension (n = 13)						
CKD vs. no CKD (eGFR ≥ 60 mL/min/1.73 m^2^)	6.20 (2.02–19.03)	0.001	23.19 (2.26–237.84)	0.008	3.51 (0.87–14.10)	0.077
Syncope (n = 11)						
Current smoking vs. no smoking	4.54 (1.17–17.57)	0.029	9.76 (0.92–103.95)	0.059	2.12 (0.34–13.16)	0.419
Obese vs. normo/underweight	0.31 (0.08–1.26)	0.102	n/a	0.958	22.87 (2.13–245.85)	0.010
Electrolyte abnormality (n = 34)						
Overweight vs. normo/underweight	0.51 (0.21–1.27)	0.148	1.14 (0.21–6.21)	0.880	0.28 (0.09–0.89)	0.031
Obese vs. normo/underweight	0.33 (0.14–0.77)	0.010	0.66 (0.11–3.85)	0.648	0.25 (0.09–0.65)	0.005
Injurious fall (n = 12)						
CKD vs. no CKD (eGFR ≥ 60 mL/min/1.73 m2)	2.44 (0.77–7.76)	0.129	n/a	0.949	4.78 (1.23–18.58)	0.024
Acute kidney injury or acute renal failure (n = 27)						
Black vs. nonblack ethnicity	4.02 (1.51–10.74)	0.005	3.84 (0.72–20.59)	0.117	4.26 (1.20–15.18)	0.025
Former smoking vs. no smoking	3.80 (1.47–9.85)	0.006	1.41 (0.30–6.62)	0.665	11.27 (2.37–53.60)	0.002
Current smoking vs. no smoking	3.08 (1.13–8.35)	0.027	1.11 (0.19–6.48)	0.904	6.89 (1.39–34.20)	0.018
Systolic blood pressure	1.01 (0.99–1.04)	0.175	0.97 (0.91–1.03)	0.266	1.03 (1.01–1.05)	0.029
CKD vs. no CKD (eGFR ≥ 60 mL/min/1.73 m^2^)	9.14 (4.05–20.63)	0.000	n/a	0.906	4.78 (1.75–13.03)	0.002

CKD: Chronic kidney disease; eGFR: Estimated glomerular filtration rate.

In terms of treatment-related SAEs, a consistently significant effect was shown across groups for the presence of CKD towards more events. Black vs. nonblack women and current smokers vs. nonsmokers developed significantly more related SAEs overall (HR: 5.44, 95% CI: 1.59–18.60 and HR: 3.55, 95% CI: 1.22–10.30; respectively), an effect not observed in the standard- or intensive-treatment group (Table 3).

The risk of serious hypotension was elevated in patients with vs. without CKD (HR: 6.20, 95% CI: 2.02–19.03), which was also observed in the standard-treatment group. No parameter was found to predict a serious bradycardia event in either treatment groups or overall (Table 3).

While serious syncope events developed more in current smokers vs. nonsmokers (HR: 4.54, 95% CI: 1.17–17.57) overall; the risk was also significantly higher in obese vs. normo/underweight women in the intensive-treatment arm (HR: 22.87, 95% CI: 2.13–245.85). On the other hand, injurious falls occurred significantly more in patients with vs. without CKD only in the intensive-treatment group (HR: 4.78, 95% CI: 1.23–18.58). Serious electrolyte abnormalities developed less among overweight or obese vs. normo/underweight women, which was especially marked in the intensive-treatment group (HR: 0.28, 95% CI: 0.09–0.89 and HR: 0.25, 95% CI: 0.09–0.65, respectively), (Table 3). 

The risk of acute kidney injury or renal failure SAE was significantly increased among black population (HR: 4.02, 95% CI: 1.51–10.74), in former (HR: 3.80, 95% CI: 1.47–9.85) or current smokers (HR: 3.08, 95% CI: 1.13–8.35), and in women with CKD (HR: 9.14, 95% CI: 4.05–20.63); which were also shown in the intensive-treatment arm compared to those in the standard-treatment arm. In addition, the risk was also significantly higher with elevated basal SBP only in the intensive-treatment group (HR: 1.03, 95% CI: 1.01–1.05), (Table 3). 

## 4. Discussion

This study is expected to contribute to the treatment approach of middle-aged hypertensive women which seems to represent a comparably gray zone. The subanalysis of the SPRINT in <65-year-old female population has featured several baseline characteristics for the follow-up of hypertension, managed with either standard or intensive approach: smoking, aspirin use, CKD, hyperglycemia, and the age for the risk of clinical outcomes and SAEs; black race and BMI only for SAEs. These key characteristics seem to be associated with several outcomes or events distinctively, which may depend on selection of the standard- or intensive-treatment strategy. 

A well-established risk factor for CV disease [10], smoking was reported to affect women more adversely than men [1,19]. Furthermore, a study showed the largest gap between smoking women and men compared to their never-smoking counterparts in terms of increased MI risk occurred between ages of 55–64 years [20]. Consistently, our subgroup analysis in middle-aged women also showed current smoking to be associated with higher risk for the primary outcome, any death, and several SAEs (any, related, syncope, and acute kidney injury/failure). Indeed, though less pronounced, the risk was also partially relevant for former smokers vs. nonsmokers, supporting the reduced CV disease risk upon smoking cessation [21]. On the other hand, association of active smoking at baseline with increased CV risk was only significant in the intensive-treatment group, suggesting a failure of the strategies targeting SBP goal <120 mmHg among active smoker vs. nonsmoker middle-aged hypertensive women. In fact, a recent analysis of SPRINT and ACCORD data reported current smokers to benefit less from the intensive treatment [22]. 

In overall study population, aspirin use at randomization was not associated with the primary outcome. Recently, three consecutive studies have reported that aspirin treatment had only limited and costly preventive effect, if any, in patients with increased CV risks [23–25]. Among these studies, ARRIVE study involved patients comparable to our population, reporting no significant benefit of aspirin in nondiabetic, >60-year-old cases with no interaction by age or sex [23]. Therefore, it may be suggested that aspirin treatment in <65-year-old women, a comparably lower risk group indeed [26], would not be expected to have a substantial preventive effect. On the other hand, the significantly higher risk of the primary outcome and MI among aspirin users in the intensive-treatment arm could have clinical importance. The presence of such an aspirin-based risk gap merely in this group implies that the decision towards intensive treatment in middle-aged hypertensive women should be reevaluated in case that they receive aspirin for a particular indication. Though we did not have data about the purpose of aspirin therapy -primary or secondary-, its concomitance with intensive BP-lowering strategy appears to worsen the outcomes, while it merits further research to elucidate underlying mechanisms or contributing factors.

Not just an independent risk factor for CV disease, CKD further seems to compel benefit/risk ratio of BP-lowering therapy in terms of target goals [27]. We observed a positive association of CKD to the increased risk of heart failure near 6.5-fold, and to lesser but significantly raised risks of total mortality, composite endpoints, and several SAEs in this middle-aged hypertensive women population. While this pattern was mainly preserved in the standard-treatment group, CKD in the intensive-treatment group was not associated with increased risk of clinical or safety outcomes, except treatment-related and acute kidney injury/failure SAEs. The difference in favor of intensive strategy is consistent with meta-analyses and CKD subanalysis of the SPRINT [28,29], confirming the benefits of such approach also in middle-aged hypertensive women with CKD. Furthermore, a recent subanalysis reported that most of such acute kidney injury events are mild in nature and completely resolved [30], which may help to relieve such potential concerns. It might be suggested that intensive approach could be encouraged in this patient subgroup accompanying CKD.

The incidence of hypertension and its associated mortality is higher in blacks vs. whites [6]. Indeed, African American postmenopausal women in Women’s Health Initiative study were reported to have lowest rate of ideal CV health [31]. While black race was associated with greater CV benefit of intensive treatment in the SPRINT overall [22], it did not predict clinical outcomes in our subanalysis of middle-aged women. By contrast, being a <65-year-old hypertensive black women was associated with increased risks of any, related, and acute kidney injury/failure SAEs in our study. Especially the latter may be attributed to the underlying etiology of racial disparities in hypertension, involving reduced ability to excrete sodium loading and different renin-angiotensin-aldosterone system in blacks [32]. In addition, >50-year-old black women were reported to be far more beyond their recommended threshold level of sodium intake than were their white counterparts despite similar amount of consumption [33]. Whether this may contribute to increased SAEs among black women warrants further research.

Age was associated with a lower risk of heart failure in the intensive-treatment group. On the contrary, a study on young adult heart failure patients reported similar rates of underlying hypertension and comparable use of antihypertensive drugs between 50–59- and 60–69-years’ age groups, suggesting no age-related association [34]. This is likely to indicate the benefit of selecting intensive antihypertensive strategy to prevent heart failure in middle-aged women as their age increases. It is further enhanced by the absence of increased risk of any SAEs in the intensive-treatment group, unlike the standard-treatment group. While older age is expected to be correlated with higher SAE rates [35,36], no association of age to the treatment-related SAEs may help to attenuate potential age-based SAE concerns, if any, in <65-year-old hypertensive women population.

Despite being a well-known risk factor for hypertension [37], obesity did not predict clinical outcomes in <65-year-old hypertensive women in this study. By contrast, it appeared to be negatively associated with serious electrolyte abnormalities and positively associated with syncope particularly in the intensive-treatment group. The latter could be partly attributed to the potent reversal of increased sympathetic nervous system activity, an important mechanism of hypertension in obese patients [38]. Whatever the reason, it may be advised for physicians managing obese hypertensive women to pay more attention in accurate BP measurement when considering intensive strategy. This is based on the fact that erroneous measurements due to mismatched cuff size in obese people, still a common practice, lead to overestimated readings and consequently unnecessary treatment [39]. This, in turn, may translate into overshooting of <120 mmHg target, aggravating already raised syncope risk. On the other hand, decreased risk of serious electrolyte abnormalities could be related with expanded intravascular volume among obese hypertensive patients [40] and elevated plasma solute concentrations in obese people [41]. This may imply a higher tolerability to clinically relevant electrolyte imbalances by antihypertensive medication. Considering the CV disease burden caused by obesity, it may be suggested that its relative protective role against electrolyte abnormalities not be overemphasized.

The positive association of baseline hyperglycemia with non-MI ACS regardless of antihypertensive strategy should be evaluated in caution. While a recent study reported higher CV or coronary mortality in ever prediabetic patients than normoglycemic patients [42], the lack of such impact of hyperglycemia on other clinical outcomes, i.e. being confined to unstable angina mostly, seem to call the finding in question. In addition, any deterioration in glycemic status during the study might have a confounding effect.

More patients with high TC/HDL-C ratio existed in the intensive-arm at baseline, yet this did not reflect as difference in clinical outcomes. Potentially influenced by several factors such as dietary habits, physical exercise, or lipid-lowering drugs [43], TC/HDL-C ratio does not appear as a good predictor to determine either standard or intensive therapy in middle-aged hypertensive women.

Polypharmacy is well-recognized risk factor for adverse effects [44]. This, at first sight, imply increased risk of adverse effects in the intensive group. However, SAE rates did not differ between treatment arms, suggesting that it is advisable not to overvalue the role of SAEs in selecting antihypertensive strategy in favor of or against to intensive therapy.

Our subanalysis has several limitations. First, in this middle-aged women population, a 3.25-year median follow-up could be regarded as a comparably shorter period to disclose the differences between treatment arms in terms of clinical outcomes. This might also reflect into the predictability of examined variables, as evident by broad CIs in several outcomes where fewer events occurred. Second, rather than being based on individual and single measurements, BP assessments were made with respect to the intended SBP target of the SPRINT, <140 or <120 mmHg. In addition, study design did not force participating clinicians in favor of any antihypertensive class or dose as long as they were guideline-based. Therefore, individual BP recordings and antihypertensive medications and their doses might influence the impact of some risk factors at individual basis. However, it could be suggested that this effect is unlikely to be that much pronounced as the study design applied for both of the treatment arms. Third, there was no baseline data about the use of hormone replacement therapy (HRT) in this subgroup. Though evidence showed that HRT resulted only small BP changes [45,46]; there is possibility that HRT could interact with particular antihypertensive medications that might influence future outcomes. On the other hand, menopausal transition was reported not to substantially act on CV outcomes [8]. In fact, the risk was suggested to depend on patients’ lifestyles rather than their hormone profile [47]. Finally, it is important to note that, the examined variables were recorded at the beginning of the study as baseline characteristics and the possible changes during the follow-up period were not evaluated.

In conclusion, smoking, aspirin use, CKD, hyperglycemia, black race, age, and BMI seemed as important baseline characteristics in the follow-up of <65-year-old hypertensive women, also depending on the therapeutic strategy. Consistent with total CV risk assessment concept, the success of antihypertensive treatment in daily practice –not confined to only BP values- is enhanced by on-point and rational consideration of suitable baseline parameters of patient subsets. This is especially relevant for populations underrepresented in trials, where subgroup analyses like the current one, aim to shed light on. In this context, such subanalyses could help to customize identify and measure the impact of associated baseline factors in particular patient populations. Therefore, abovementioned baseline parameters should be considered for a rational antihypertensive management, including treatment and follow-up, that contributes to prevent CV outcomes and avoid SAEs in this middle-aged female population with increased CV risk. With a higher life expectancy in the real-world setting, middle-aged hypertensive women might have better outcomes if intensity of BP-lowering strategy is built on predictive factors at baseline. 

## Acknowledgments

This manuscript was prepared using SPRINT_POP Research Materials obtained from the National Heart, Lung, and Blood Institute (NHLBI) Biologic Specimen and Data Repository Information Coordinating Center and does not necessarily reflect the opinions or views of the SPRINT_POP or the NHLBI.

## Funding

This subanalysis did not receive any specific grant from funding agencies in the public, commercial, or not-for-profit sectors.

## Informed consent

The subanalysis was performed after signing data-sharing protocol with SPRINT investigators based on the study approval from local institutional ethics committee (Approval no. 09.2016.664).
